# Supplementary material for: Peritrophin-like Genes Are Associated with Delousing Drug Response and Sensitivity in the Sea Louse Caligus rogercresseyi
Source: Int J Mol Sci. 2022 Nov 1;23(21):13341. doi: 10.3390/ijms232113341 (PMC9655413; doi:10.3390/ijms232113341)
Supplement: Supplementary file 1 [file ijms-23-13341-s001.zip › Supplementary Figure.pdf]

Supplementary figures

Figure S1. Prediction of the three-dimensional model of peritrophin-like protein from *C. rogercresseyi*. In parentheses, it shows the secondary conformations percentages in the sequences (α-helix/ β-sheet). **A.** Cr_Per1, **B.** Cr_Per3, **C.** Cr_Per4, **D.** Cr_Per7.

Figure S2. Schematic representation of Cr_Per5 gene organization. The brown boxes represent the coding sequences, the salmon box represents the intron whose sequence is amplified. The orange hexagons represent the ChBD2 domains of the protein and the purple oval a region of low complexity.

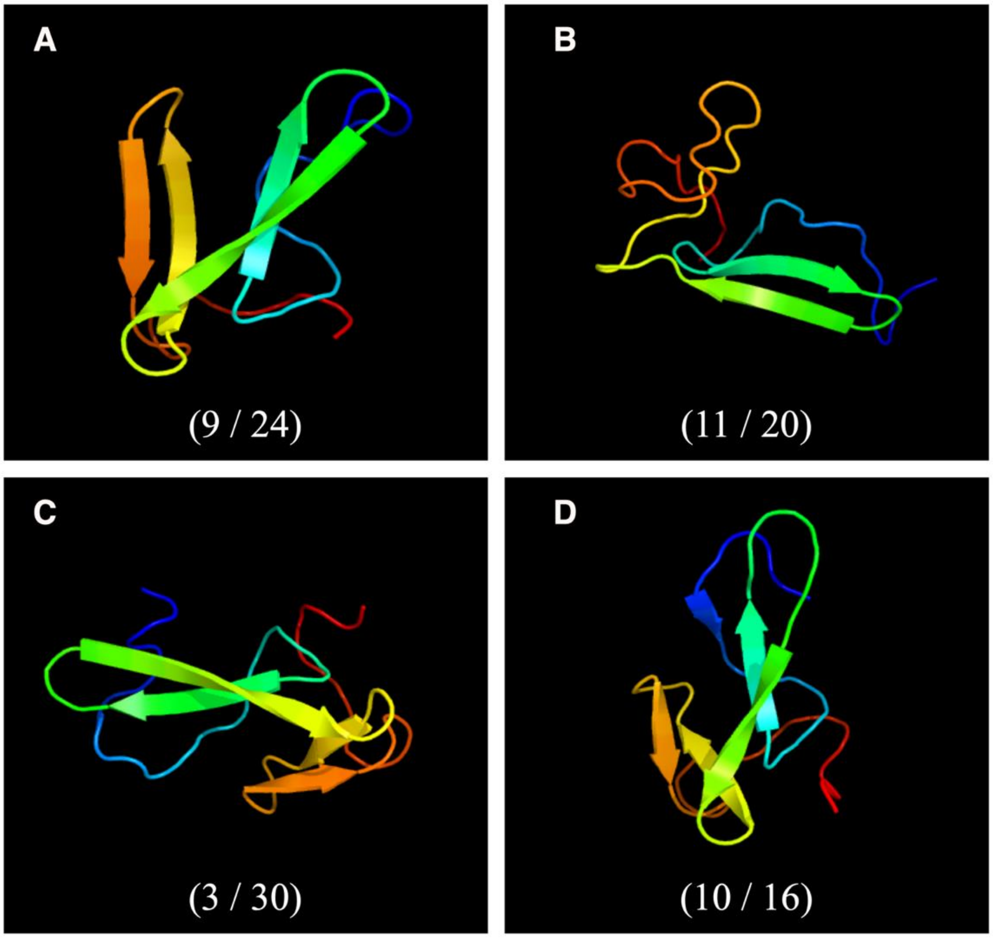

Figure S1

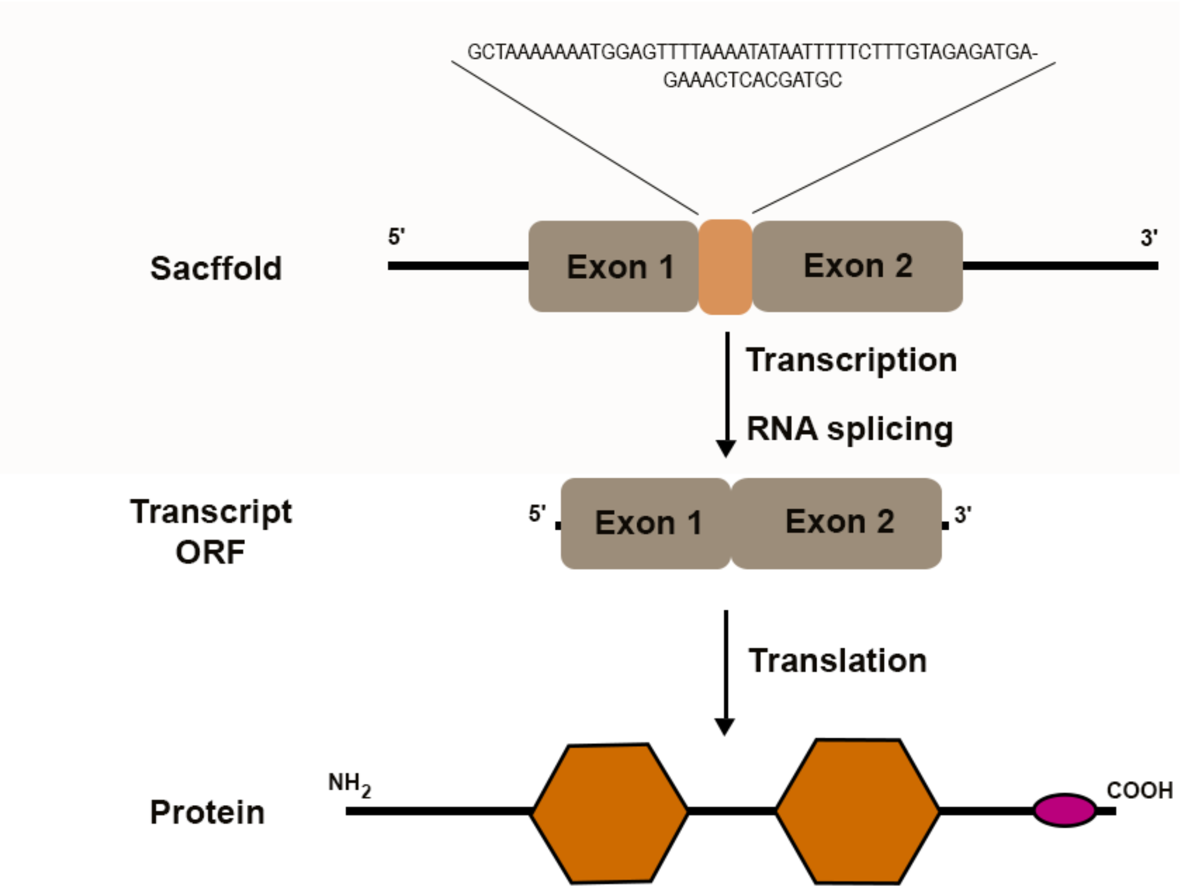
GCTAAAAAAAATGGAGTTTTAAAATATAATTTTTCTTTGTAGAGATGA-
GAAACTCACGATGC
Sacffold
5'
Exon 1
Exon 2
3'
Transcription
RNA splicing
Transcript
ORF
5'
Exon 1
Exon 2
3'
Translation
Protein
NH2
COOH

Figure S2
