# Supplementary material for: Peritrophin-like Genes Are Associated with Delousing Drug Response and Sensitivity in the Sea Louse Caligus rogercresseyi
Source: Int J Mol Sci. 2022 Nov 1;23(21):13341. doi: 10.3390/ijms232113341 (PMC9655413; doi:10.3390/ijms232113341)
Supplement: Supplementary file 1 [file ijms-23-13341-s001.zip › Supplementary Table.pdf]

Table S1. Contigs annotated as *C. rogercresseyi* peritrophin-like gene isoform.

Gene	Description	Number of HSPs	E-value	Accession
contig10	Peritrophin-1 [*Lepeophtheirus salmonis*]	13	6,16E-144	ADD24486
contig12860	Peritrophin-1 precursor [*Caligus clemensi*]	11	2,00E-138	ACO14568
contig12862	Peritrophin-1 precursor [*Caligus clemensi*]	12	1,53E-124	ACO14568
contig6390	Peritrophin-1 [*Lepeophtheirus salmonis*]	12	5,31E-69	ADD24486
contig4172	Peritrophin-1 [*Lepeophtheirus salmonis*]	6	3,86E-42	ADD38464
contig6396	Peritrophin-1 [*Lepeophtheirus salmonis*]	11	7,08E-37	ADD24486
contig8519	Peritrophin-1 precursor [Caligus clemensi].	11	1,58E-34	ACO14568
contig4785	Peritrophin-1 [Lepeophtheirus salmonis].	6	2,25E-07	ADD38464
contig4784	Peritrophin-1 [Lepeophtheirus salmonis].	5	2,48E-07	ADD38464
Contig0008088	TSA: CPAP [Lepeophtheirus salmonis]	13	7,53E-169	HACA01011048
Contig0074192	TSA: CPAP [Lepeophtheirus salmonis]	14	1,53E-117	HACA01011048
Contig0002090	CPAP [Tribolium castaneum]	11	2,44E-101	NM_001080099
Contig0024461	TSA: CPAP [Lepeophtheirus salmonis]	13	3,78E-101	HACA01028014
Contig0003224	TSA: partial CPAP [Lepeophtheirus salmonis]	9	1,67E-99	HACA01018620
Contig0016844	TSA: CPAP [Lepeophtheirus salmonis]	14	6,27E-99	HACA01011048
Contig0010845	TSA: CPAP [Lepeophtheirus salmonis]	18	1,39E-95	HACA01011048

CPAP: Cuticular Protein Analogous to Peritrophins
